# Genetic studies in clonal haematopoiesis, myelodysplastic neoplasms and acute myeloid leukaemia – a practical guide to WHO-HAEM5

**DOI:** 10.1515/medgen-2024-2010

**Published:** 2024-03-06

**Authors:** Katharina Hörst, Constanze Kühn, Claudia Haferlach, Torsten Haferlach, Joseph D. Khoury

**Affiliations:** MLL – Munich Leukemia Laboratory Munich Germany; MLL – Munich Leukemia Laboratory Munich Germany; MLL – Munich Leukemia Laboratory Munich Germany; MLL – Munich Leukemia Laboratory Munich Germany; University of Nebraska Medical Center Department of Pathology and Microbiology Omaha USA

**Keywords:** WHO, classification, clonal haematopoiesis, myelodysplastic neoplasms, acute myeloid leukaemia

## Abstract

In recent years, technology developments and increase in knowledge have led to profound changes in the diagnostics of haematologic neoplasms, particularly myeloid neoplasms. Therefore an updated, fifth edition of the World Health Organization (WHO) classification of haematolymphoid neoplasms (WHO-HAEM5) will be issued in 2024. In this context, we present a practical guide for analysing the genetic aspects of clonal haematopoiesis of indeterminate potential (CHIP), clonal cytopenia of undetermined significance (CCUS), myelodysplastic neoplasms (MDS), and acute myeloid leukaemia (AML) based on WHO-HAEM5. This guide navigates through the genetic abnormalities underlying myeloid neoplasms which are required to be detected for classification according to WHO-HAEM5 and provides diagnostic algorithms.

## From CHIP to CCUS to MDS and AML: it’s a continuum

Ground-breaking advances in high-throughput genome sequencing technologies have not only deepened our insights into the pathobiology of myeloid neoplasms, but have also revealed ways to revolutionize diagnostics and therapeutic strategies.

The journey towards understanding clonal haematopoiesis of indeterminate potential (CHIP), clonal cytopenia of undetermined significance (CCUS), myelodysplastic neoplasms (MDS) and acute myeloid leukaemia (AML) has witnessed a paradigm shift in recent times. For the first time, WHO-HAEM5 includes clonal haematopoiesis (CH), the detection of clonality in haematopoietic cells, as a myeloid precursor lesion [1, 2]. In CH, CHIP is differentiated from CCUS – together with MDS and AML these conditions are now recognized as interlinked components of a dynamic disease continuum, marked by a discernible pre-malignant phase [1]. A better understanding of the underlying genetics has revealed mutations with diagnostic and prognostic value. In addition, known mutations provide tools to explore clonal diversity and disease progression.

## Clonal haematopoiesis of indeterminate potential (CHIP)

Extensive exome sequencing studies on more than 30,000 individuals without known haematological disorders led to the detection of somatic mutations within blood DNA with increasing frequency in patients aged 65 or older [3–5]. Clonal haematopoiesis is present in 10–40 % of older individuals and is rarely detected in individuals under the age of 40 years. However, the prevalence depends on the sensitivity of the diagnostic sequencing method and the number of genes analysed [1, 6]. Using techniques with very high sensitivity and a large number of genes clonal haematopoiesis can be detected in the majority of people. To encapsulate this phenomenon, the term CHIP (Clonal Haematopoiesis of Indeterminate Potential) was coined and a minimum variant allele frequency (VAF) and a distinct set of genes defined (see below). Most individuals exhibiting mutations displayed a solitary mutation. Interestingly, three genes – *DNMT3A*, *TET2*, and *ASXL1* – accounted for the majority of driver mutations sustaining clonal haematopoiesis [3].

A CHIP diagnosis necessitates detecting somatic mutations in myeloid malignancy-associated genes in the absence of unexplained cytopenia or diagnosed haematologic disorder [1].

### CHIP: definition

Within WHO-HAEM5, CHIP is defined by the following criteria [1]:

Detection of one or more somatic mutations with a variant allele frequency (VAF) of 2 % or more (4 % or more for X-linked gene mutations in males) in the DNA of blood or bone marrow cells involving defined genes
Absence of unexplained cytopenias
Absence of diagnostic criteria for defined myeloid neoplasms

### CHIP diagnosis today is typically an incidental finding

Molecular profiling of peripheral blood in individuals without abnormalities in the blood count in a different medical context such as liquid biopsy for solid tumours can lead to the detection of CHIP.

#### CHIP: cytomorphology and cytogenetics

If somatic mutation profiling meets the criteria of CHIP, a blood count analysis including cytomorphological examination should be performed to differentiate CHIP from CCUS or a haematologic neoplasm. Further diagnostic testing like bone marrow aspiration is indicated if haematologic neoplasia is suspected [7].

Chromosome analysis is not indicated in CHIP diagnosis but should be performed on the bone marrow if unexplained cytopenia is present.

#### CHIP: molecular genetics

As defined by WHO, evidence of clonality in bone marrow or blood cells must be provided [1]. This can be done by the detection of somatic mutations, typically by DNA sequencing of blood cells. Furthermore, the selected diagnostic method must be able to detect a VAF of 2 % reliably [6]. According to the WHO-HAEM5, CHIP requires the presence of at least one mutation in any of the following genes [1]:

Common/clinically significant genes: *DNMT3A, TET2, ASXL1, JAK2, TP53, SF3B1, PPM1D, SRSF2, IDH1, IDH2, U2AF1, KRAS, NRAS, CTCF, CBL, GNB1, BRCC3, PTPN11, GNAS, BCOR, BCORL1*
Additional genes to be analysed: *BRAF, CALR, CEBPA, CRBBP, CSF1R, CSF3R, CUX1, ETV6, EZH2, GATA2, JAK3, KDM6A, KIT, KMT2A, MPL, MYD88, NOTCH1, PHF6, PIGA, PRPF40B, PTEN, RAD21, RUNX1, SETBP1, SF1, SF3A1, SMC1A, SMC3, STAG2, STAT3, U2AF2, WT1, ZRSR2*

The annual risk of transformation in CHIP carriers is lower than initially assumed (0.17–0.22 % instead of 1 %) [6]. However, factors such as the presence of a large clone or multiple gene mutations are associated with an increased risk of progression to myeloid malignancy [1].

Apart from its risk of progressing to myeloid neoplasia, CHIP is associated with an elevated susceptibility to cardiovascular disease. Remarkably, CHIP’s impact as a risk factor matches established cardiovascular risk factors like smoking. Indeed, CHIP carriers exhibit an augmented risk of coronary heart disease [8, 9]. Furthermore, CH was also found to be linked to an increased risk of overall lethality and non-haematologic diseases including liver disease, solid cancers, chronic obstructive pulmonary disease, and gout [10]. Thus, the interest in appropriate diagnostics for CHIP is gaining overall importance due to its clinically relevant consequences [7].

## Clonal cytopenia of undetermined significance (CCUS)

With regard to the diagnostic criteria, CCUS is defined analogously to CHIP, but in the case of CCUS one or more otherwise unexplained cytopenias persisting for more than four months have to be present. CCUS is associated with an increased risk of developing haematologic neoplasia, especially with an allele burden of ≥10 % or if more than one mutation is detected [1, 6]. To facilitate improved progression risk prediction for clonal haematopoiesis, the clonal haematopoiesis risk score (CHRS) was recently established. By considering parameters such as high-risk mutations, clone size, cytopenia, and age, the CHRS assigns patients into low-risk, intermediate-risk and high-risk groups [11].

CCUS and MDS have cytopenia(s) and clonal alterations in common, but in CCUS the morphologic diagnostic criteria for MDS are not met [1].

## Myelodysplastic neoplasms (MDS)

WHO-HAEM5 introduced the term myelodysplastic *neoplasms* (MDS) to replace myelodysplastic *syndromes,* thereby emphasizing their neoplastic nature and harmonizing terminology with myeloproliferative neoplasms (MPN) [1].

Differentiation between CHIP, CCUS and MDS cannot solely rely on molecular genetic testing but currently depends on the presence of cytopenias and morphologic diagnostic criteria specific to MDS. The most significant differences between CHIP and MDS are the lack of dysplasia and cytopenia [1]. The transition among CHIP, CCUS, and MDS is presumed to occur seamlessly through clonal evolution and selection. This assumption is supported by the increase in genetic complexity (as shown in Table 1), characterized by an increase in the number of mutations and the size of the clonal population (allele frequency) [12–15].

**Table 1:** Genetic characteristics of CHIP, CCUS and MDS [1, 12, 16]

	CHIP	CCUS (at diagnosis)	MDS (all risk groups)
Cytopenia [16]	–	+	+
Dysplasia [16]	–	–	+
Blasts [1]	<5 %	<5 %	<20 %
Frequently mutated genes [12]	*DNMT3A, TET2, ASXL1, PPM1D, JAK2, TP53*	*TET2, DNMT3A, ASXL1, SRSF2, TP53*	*SF3B1, TET2, ASXL1, SRSF2, DNMT3A*
Average number of mutated genes [12]	~1	~1.6	~2.6
Typical allele frequency [12]	9–12 %	30–40 %	30–50 %

**Figure 1:**
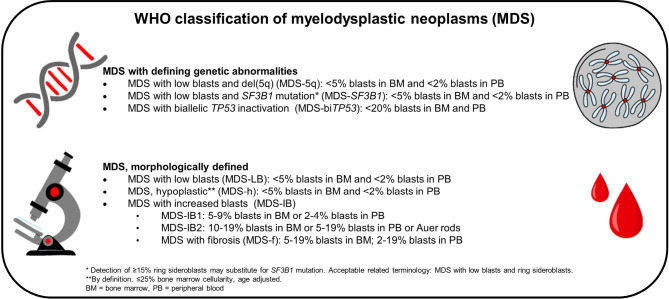
WHO classification of myelodysplastic neoplasms (MDS)

The WHO classification provides a clear distinction between MDS with specific genetic abnormalities and MDS defined solely by morphological characteristics (Figure 1). For a diagnosis of MDS according to WHO, cytopenia in at least one haematopoietic lineage is required (anaemia (haemoglobin <13g/dL in men or <12g/dL in women), neutropenia (absolute neutrophil count <1.8 × 10^9/^L) and/or thrombocytopenia (platelets <150 × 10^9/^L) [1].

### MDS: cytomorphology

Morphologically, dysplasia in one or more myeloid lineages is the hallmark of MDS. Further, assessing the blast count (<5 %, 5–9 %, 10 – <20 %) is required for MDS classification and distinction from AML (≥20 %) [1].

### Diagnostics of MDS: three genetic subtypes have been defined

Genetic testing encompassing karyotyping and mutation screening is a central component of MDS classification [1].

#### MDS: cytogenetics

At diagnosis, approximately 50 % of MDS patients exhibit clonal chromosome aberrations. Around 11 % of patients demonstrate multiple abnormalities, constituting complex karyotypes (≥3 or more aberrations) [17].

Predominant karyotypic anomalies in MDS include del(5q), del(7q) or monosomy 7 (–7), trisomy 8 (+8), del(20q), and loss of Y chromosome. Deletion of the long arm of chromosome 5 is the most frequent chromosomal aberration in MDS, even constituting its own entity within the WHO classification if certain criteria are met (see below) [1]. Thus, the karyotype is required for the classification of MDS according to WHO-HAEM5 and also for prognostication. For many years, the International Prognostic Scoring System (IPSS) [18] was the mainstay of the prognostic classification in patients with MDS. For improved and more detailed risk stratification of patients with MDS, the IPSS was revised in 2012 (Revised-IPSS, IPSS-R) [19]. The contemporary strong emphasis on molecular genetics is reflected in a new prognostic score (IPSS-M), which takes molecular genetic findings into account in addition to established clinical and cytogenetic categories [20].

Myelodysplastic neoplasm with low blasts and 5q deletion (MDS-5q) is a myeloid neoplasm defined by the following criteria [1]:

Anaemia, with or without other cytopenias and/or thrombocytosis;
Dysplasia involving megakaryocytes, with or without dysplasia involving other lineages;
Blasts <5 % in the bone marrow and <2 % in the peripheral blood;
Detection of 5q deletion, isolated or with one other cytogenetic aberration other than monosomy 7 or 7q deletion;
Not fulfilling diagnostic criteria of AML, MDS with biallelic *TP53* inactivation, MDS with increased blasts, or MDS/MPN

#### MDS: molecular genetics

Several studies have revealed the landscape of somatic point mutations in MDS using panels of genes previously implicated in myeloid disorders. Up to 90 % of patients have mutations in at least one recurrently altered gene, while the remaining 10 % may carry as yet unidentified gene mutations [21, 22].

The importance of molecular genetics in MDS is reflected in the WHO classification that distinguishes, in addition to MDS-5q, two further subtypes according to their genetic aberrations: MDS-*SF3B1*, and MDS-bi*TP53* [1].

Myelodysplastic neoplasm with low blasts and *SF3B1* mutation (MDS-*SF3B1*) is a myeloid neoplasm characterized by the following criteria [1]:

Cytopenia involving one or more lineages, without thrombocytosis;
Erythroid lineage dysplasia;
Blasts <5 % in the bone marrow and <2 % in the peripheral blood;
Detection of *SF3B1* mutation. If *SF3B1* mutation analysis is not available, demonstration of ring sideroblasts comprising ≥15 % of erythroid precursors;
Absence of 5q deletion, monosomy 7/7q deletion, or complex karyotype.
Not fulfilling diagnostic criteria of AML, MDS with low blasts and 5q deletion, MDS with biallelic *TP53* inactivation, MDS with increased blasts, or any MDS/MPN type.

MDS-*SF3B1* has the best outcome among MDS types [1]. It is a myeloid neoplasm with cytopenia and dysplasia characterized by *SF3B1* mutation and often ring sideroblasts. Identification of typically heterozygous *SF3B1* mutations, usually at high VAF (median 35–43 %), is required for diagnosis [1]. Certain co-mutations, such as *BCOR, BCORL1, NRAS, RUNX1, SRSF2* or *STAG2*, have been recently correlated with adverse impact in MDS-*SF3B1* showing significantly different outcomes in comparison to mutation in *SF3B1* alone [20].

*SF3B1* mutations can be detected in other MDS subtypes or other myeloid neoplasms. Due to the hierarchical order of WHO-HAEM5 if the criteria for MDS with low blasts and 5q deletion are fulfilled, cases should be classified as such, even if a *SF3B1* mutation was identified [1].

Myelodysplastic neoplasm with biallelic (or multi-hit) *TP53* alterations (MDS-bi*TP53*) is defined by the following criteria [1]:

Myeloid neoplasm fulfilling diagnostic criteria of MDS
Detection of one or more *TP53* mutations
In the presence of one *TP53* mutation, evidence of *TP53* copy loss or copy neutral loss of heterozygosity (LOH)

Biallelic *TP53* alterations appear to drive myeloid neoplasms by different mechanisms than monoallelic ones: MDS-bi*TP53* cases have significantly less additional driver mutations and higher numbers of copy number abnormalities and cytogenetic lesions. This “multi-hit” mutational status results in a neoplastic clone without any wild-type p53 protein. Diagnostics requires sequencing analysis as well as the detection of the copy number status. For this, fluorescence in situ hybridisation (FISH) with a probe set specific for the *TP53* locus on 17p13.1 is typically used [1].

Along the disease continuum, MDS is associated with an increased risk of transformation to AML [1, 20].

## Acute myeloid leukaemia (AML)

**Figure 2:**
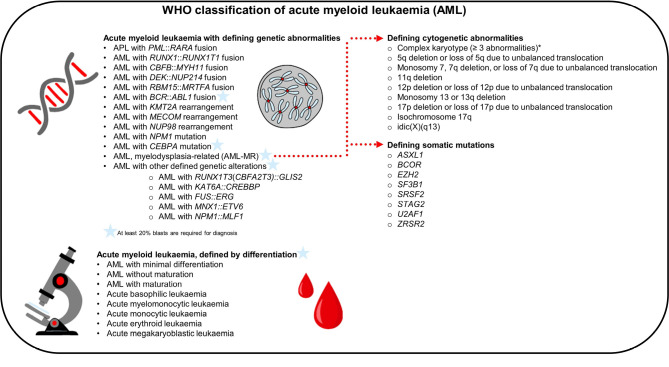
WHO classification of acute myeloid leukaemia (AML)

Acute myeloid leukaemia (AML) may arise *de novo*, or secondarily after prior cytotoxic chemotherapy and/or radiotherapy (AML-pCT) or preexisting myelodysplastic/myeloproliferative neoplasm or MDS (s-AML). The WHO classification divides AML into ‘AML with defining genetic abnormalities’ and ‘AML, defined by differentiation’, the former encompassing gene fusions, rearrangements, and mutations (Fig. 2) [1].

### Diagnostics in AML is based on phenotype and genotype

In AML diagnostics, cytomorphology, immunophenotyping, cytogenetics, and molecular genetics are required for classification and prognostication [23].

#### AML: cytomorphology and immunophenotyping

Cytomorphology, augmented by cytochemistry, holds a fundamental position in confirming AML diagnoses and facilitating their classification as per WHO guidelines. In the absence of defining genetic aberrations, the classification of the AML subtypes “defined by differentiation” takes place based on cytomorphology. Its utility extends to monitoring remission during therapy, thereby maintaining its status as the gold standard for follow-up assessments.

Immunophenotyping plays a critical role in routine AML diagnosis, elucidating disease heterogeneity. Pertinent markers, such as HLA-DR and CD34 (negative) in acute promyelocytic leukaemia (APL), CD19 and CD56 in AML with maturation and AML with *RUNX1*::*RUNX1T1* fusion, and CD2, CD15, and CD34 in myelomonocytic AML with abnormal eosinophils, provide insights essential for precise subclassification and informed clinical management [1].

#### AML: cytogenetics

Chromosome analysis is currently the gold standard to detect structural and numerical chromosome aberrations. This information is required for classification according to WHO standards, but also for prognostication according to the current European LeukemiaNet (ELN) guidelines that can have a significant influence on patient treatment strategies [23, 24].

If conventional cytogenetic analysis fails, FISH can be used as an alternative for the detection of fusions and rearrangements like *RUNX1*::*RUNX1T1*, *CBFB*::*MYH11*, and rearrangements of *KMT2A* (*MLL*), and *MECOM* (*EVI1*) [23].

In general, for a diagnosis of AML a blast count of at least 20 % is required. However, certain genetic abnormalities have long been accepted as AML-defining, thereby negating the need for this criterion. In WHO-HAEM5, the number of AML-defining genetic abnormalities that negate this blast requirement have increased further (see also Fig. 2) [1, 25]:

**APL with** ***PML*::*RARA* fusion**: Resulting from t(15;17)(q24;q21), it causes acute promyelocytic leukaemia, necessitating immediate treatment. The prognosis is excellent with prompt ATRA and/or arsenic trioxide therapy.
**AML with** ***RUNX1*::*RUNX1T1* fusion**: The t(8;21)(q22;q22.1) is associated with a favourable prognosis in adults but relatively poor prognosis in children, with specific genetic context influencing leukemic development and progression.
**AML with** ***CBFB*::*MYH11* fusion**: Typically due to inv(16)(p13.1q22) or t(16;16)(p13.1;q22), found in younger patients, it shows favourable prognosis and good responses to therapy.
**AML with** ***DEK*::*NUP214* fusion**: t(6;9)(p22.3;q34.1), present in a minority, patients show poor response to standard therapy, with frequent *FLT3*-ITD occurrence.
**AML with** ***RBM15*::*MRTFA* fusion**: Occurs rarely, primarily in infants and young children.
**AML with** ***KMT2A* rearrangement**: Common in children, prognosis varies with fusion partner.
**AML with** ***MECOM* rearrangement**: Overexpression of *EVI1* is caused by the translocated partner gene enhancer in rearrangements of the *MECOM* locus, frequently complex karyotype, aggressive course and unfavourable prognosis.
**AML with** ***NUP98* rearrangement**: Wide fusion partner variability, typically poor prognosis, worse with *FLT3*-ITD presence.

The diagnosis of the following two AML types still requires a bone marrow blast percentage of at least 20 %:

**AML with** ***BCR*::*ABL1* fusion**: Rare subtype, poor response to AML chemotherapy, limited benefit from tyrosine kinase inhibitors. A blast count of at least 20 % was kept to differentiate AML with *BCR*::*ABL1* fusion from chronic myeloid leukaemia (CML), although distinction from CML blast phase is still challenging, thus lack of features of CML prior to or at diagnosis or after therapy is required.
**Acute myeloid leukaemia with other defined genetic alterations**: Encompasses emerging AML subtypes with unique genetic features.

#### AML: molecular genetics

The molecular genetic diagnostics of AML has been significantly propelled by the utilization of next-generation sequencing (NGS) techniques. Introducing gene panels, the diagnostic process is streamlined, minimizing turnaround time, while simultaneously allowing a comprehensive overview of molecular aberrations with diagnostic, prognostic, and therapeutic implications [1, 23].

Entity-defining mutations in the current WHO classification are *NPM1* and *CEBPA* (see also Fig. 2). The latter includes biallelic (bi*CEBPA*) as well as single mutations located in the basic leucine zipper (bZIP) region of the gene (smbZIP-*CEBPA*). Both types are associated with a favourable prognosis [1]. *NPM1* is the only molecular mutation, if present in a myeloid neoplasm, that leads to the classification of AML irrespective of the blast count. This approach is based on data showing that cases previously classified as MDS or MDS/MPN with *NPM1* progress to AML in a short period of time [1]. However, with this change, cases previously classified as low-risk MDS are now categorised as AML. The mutational status of *NPM1* (together with *FLT3*) has to be determined for an accurate risk stratification. According to the 2022 ELN Guidelines, AML with *FLT3*-ITD is generally assigned to the intermediate risk group regardless of the allele ratio and the presence of an *NPM1* mutation. *NPM1*-mutated AML without *FLT3*-ITD, on the other hand, is assigned to the favourable risk group [1, 23].

While for AML with *NPM1* mutation no blast count threshold is defined, the blast count criterion (at least 20 %) still applies to the diagnosis of AML with *CEBPA* mutation due to insufficient available data as of now [1].

In essence, the molecular genetic diagnosis of AML relies on a combination of state-of-the-art methods such as NGS complemented by traditional PCR techniques, fragment length analysis, and quantitative real-time PCR.

Important changes in WHO-HAEM5 also apply to the AML-MR subtype (previously AML with myelodysplasia-related changes). For this subtype, morphology is no longer sufficient as the sole diagnostic criterion, cytogenetic criteria have been updated, and a mutation-based criteria has been introduced using 8 genes (listed in Fig. 2).

In addition, WHO-HAEM5 dedicates one chapter to secondary neoplasms, which are myeloid neoplasms that arise secondary to exposure to cytotoxic therapy or germline predisposition. Following WHO-HAEM5 post cytotoxic therapy and germline predisposition should be added as qualifiers to respective myeloid neoplasms, such as MDS or AML [1].

#### Measurable residual disease (MRD) in AML

The utility of measurable residual disease (MRD), the detection of low levels of residual leukemic cells, is becoming increasingly important in AML therapy management but is not yet firmly established in routine practice. However, MRD assessment in AML may be used to establish a deeper remission status and also represents a strong prognostic factor with the potential of postremission therapy guidance. The major significance of MRD in AML management is also reflected in the ELN MRD recommendations, which are regularly updated due to ongoing technological progress [24, 26].

Multiparametric flow cytometry is currently the most commonly used MRD detection tool, reaching a sensitivity of 10^-3^ to 10^-4^. Molecular MRD testing technologies, such as real-time quantitative PCR, are also extensively studied enabling a sensitivity up to 10^-4^ to 10^-5^. Not yet established for MRD detection in AML routine diagnostics but emerging exploratory technologies are digital PCR and NGS, demonstrating a detection limit of 10^-3^ to 10^-4^ and 10^-2^ to 10^-4^, respectively [23].

MRD assessment holds promise as a potential endpoint in clinical trials. The ability to accurately gauge treatment response through MRD evaluation adds an objective dimension to clinical trial outcomes. This has the potential to expedite drug development and approvals, as well as to aid in optimizing treatment regimens.

## Conclusion

**Figure 3:**
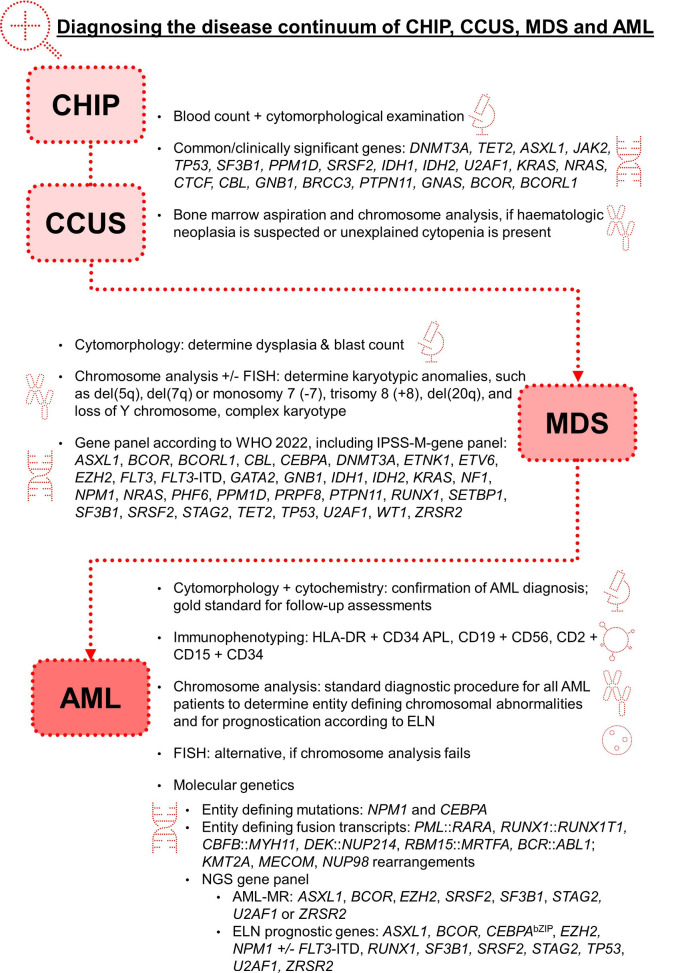
Diagnostic methods for CHIP, CCUS, MDS, and AML

Currently, CHIP, CCUS, MDS and AML are defined as distinct disease categories, but it is recognized that they represent a biologic continuum. Diagnostic algorithms need to comprehensively capture all parameters relevant for classification, prognostication and therapy decisions (see Fig. 3).

As can be seen from the diagnostics of the myeloid neoplasms presented here, genetic methods are becoming increasingly important in the diagnosis of haematologic neoplasms. However, a diagnosis solely based on genetic testing is not possible at present. All current phenotypic and genotypic laboratory methods are necessary for a comprehensive diagnosis and classification.
